# A new species of hydrobiid snails (Mollusca, Gastropoda, Hydrobiidae) from central Greece

**DOI:** 10.3897/zookeys.138.1927

**Published:** 2011-10-19

**Authors:** Canella Radea

**Affiliations:** 1 Department of Ecology and Systematics, Faculty of Biology, School of Sciences, National and Kapodistrian University of Athens, 15784 Panepistimiopolis, Greece

**Keywords:** Caenogastropoda, Hydrobiidae, *Daphniola eptalophos* sp. n., Greece

## Abstract

Anew minute valvatiform species belonging to the genus *Daphniola* Radoman, 1973, *Daphniola eptalophos* **sp. n.**, from mountain Parnassos, Greece is described. The new species has a transparent valvatiform-planispiral shell, wide and open umbilicus, grey-black pigmented soft body and head and a black penis with a small colorless outgrowth on the left side near its base. A comparative table of shell dimensions and a key to the species known for this endemic genus for Greece are provided.

## Introduction

Greece is a hot spot for hydrobioid gastropods both in terms of species richness and endemism (Glöer and Maassen 2009, Glöer et al. 2010). Hydrobioid gastropods include the family Hydrobiidae and several other families of Rissooidea that resemble these gastropods in general features (Hershler and Ponder 1998). To date, 72 hydrobioid species and subspecies belonging to 24 genera have been recorded in Greece (Bank 2006, Glöeret al. 2007; Reischütz and Reischütz 2008, Reischütz et al. 2008, Glöer and Maassen 2009, Glöer et al. 2010, Szarowska and Falniowski 2011a). It is notable that 90% of these species and subspecies and 26% of the genera are endemic for Greece.

The hydrobiid gastropods (family Hydrobiidae) of Greece have been studied by several authors during the 19^th^, 20^th^ and 21^th^ century (e.g., Westerlund 1886, Boettger 1892, Käufel 1930, Schütt 1980, Gittenberger 1982, Radoman 1983, Falniowski and Szarowska 2000, Bank 2006, Frogley and Preece 2007, Albrecht et al. 2009, Reischütz et al. 2010, Benke et al. 2011, Falniowski and Szarowska 2011, Szarowska and Falniowski 2011a); nevertheless, our knowledge still remains incomplete. The IUCN Red List of Threatened Species includes 42 hydrobiid species from Greece. One of them is classified as Extinct; 19 are classified as Critically Endangered, three as Endangered, one as Near Threatened, four as Vulnerable, eight as Data Deficient and the rest as Least Concern.

*Daphniola* Radoman, 1973 (type species *Daphniola graeca* Radoman, 1973) is an endemic genus from Greece. According to Schütt (1980), *Daphniola graeca* Radoman 1973 is a junior synonym of *Valvata exigua* Schmidt, 1856 and according to Reischutz and Sattman (1993) a junior synonym of *Valvata (Cincinna) hellenica* Westerlund, 1898.

Two of the three currently known species of this genus, namely *Daphniola exigua* (A. Schmidt 1856) and *Daphniola louisi* Falniowski & Szarowska 2000 are included in the category Endangered and Critically Endangered respectively (Radea and Falniowski 2009, Radea 2011) of the Red List mentioned above. A third taxon, *Daphniola graeca* was synonymized with *Daphniola exigua* by Falniowski et al. (2007).

Recently, Falniowski and Szarowska (2011) identified a valvatiform hydrobiid gastropod found in the Peloponnisos, Greece as *Horatia hadei* Gittenberger, 1982, a species, which currently is listed as *Islamia hadei* (Gittenberger, 1982) according to Bank (2011). This gastropod was found in a distance of 40 km from the type locality of *Horatia hadei*, which probably has been destroyed (Szarowska and Falniowski 2004, Szarowska 2006). According to the above authors, the protoconch sculpture, female reproductive organs, penis morphology and a maximum likelihood phylogenetic analysis based on COI (cytochrome oxidase subunit I) fragments of mtDNA proved that this gastropod belongs to the genus *Daphniola*. Subsequently, Falniowski and Szarowska (2011) transferred the species *hadei* from *Islamia* to *Daphniola*. However, the identification of the hydrobiid gastropod found in Peloponnisos as *Horatia hadei* was only based on the resemblance of shell shape and protoconch sculpture and it was not supported by detailed morphological, morphometric and anatomical comparisons. Consequently, this identification as well as the new combination should be carefully re-examined.

The morphology and anatomy of the genus *Daphniola* have extensively been described by Radoman (1973), Radoman (1983) and Bodonet al. (2001). Morphometric variables, soft body pigmentation, male and female genitalia are widely used for the distinction of species and subspecies of this genus (Schütt 1980, Reischütz 1984, Falniowski and Szarowska 2000, Falniowski et al. 2007, Falniowski and Szarowska 2011). According to Radoman (1973), Schütt (1980), Radoman (1983), Reischütz (1984), Reischütz (1988), Falniowski and Szarowska (2000), Bodon et al. (2001), and Falniowski et al. (2007), Falniowski and Szarowska (2011) this crenobiont genus inhabits most of mainland Greece, i.e., Peloponnesos, Attica except its easternmost part, the western part of Euboea, southeast Thessalia and east Macedonia.

Here a new *Daphniola* species is described from central Greece, i.e. Sterea Ellada, and compared with its congeners.

## Materials and methods

Specimens of a minute valvatoid hydrobiid gastropod from a spring nearby Agoriani (Eptalophos, mountain Parnassos, Sterea Ellada, Greece), were collected alive. Since population abundance of this species seems to be low in the spring where it was found only eighteen specimens were collected. Thirteen of them were stored in 70% ethanol for morphological and anatomical studies and five specimens in deep freezing for future molecular analyses.

Shell morphometric variables (namely shell height and width, aperture height and width) were measured of all specimens collected using the micrometer of a Stemi 2000-C, Zeiss stereomicroscope. The ratios of shell variables were calculated as well.

The structure of protoconch and teleoconch of the shells were studied using scanning electron microscopy (Jeol JSM-35 operating at 25 kV) after being dehydrated in a gradient of ethanol dilution series (10-100%) and finally in pure acetone, critical point dried and spray coated in gold-palladium.

Six randomly chosen specimens were dissected (four of them were found to be mature males, one mature female and one immature female).

Shells and penes were photographed with a Canon Eos 1000D digital camera attached on a stereomicroscope Stemi 2000-C, Zeiss, Germany.

Abbreviations: ZMUA, Zoological Museum, National and Kapodistrian University of Athens.

## Systematics

**Hydrobiidae Troschel, 1857**

**Genus** ***Daphniola*** **Radoman, 1973**

Type species *Daphniola graeca* Radoman, 1973

### Daphniola eptalophos sp. n.

urn:lsid:zoobank.org:act:BF2C6C3F-5EF0-4375-802D-37D5529ED3E5

http://species-id.net/wiki/Daphniola_eptalophos

**Figures 1-6.**
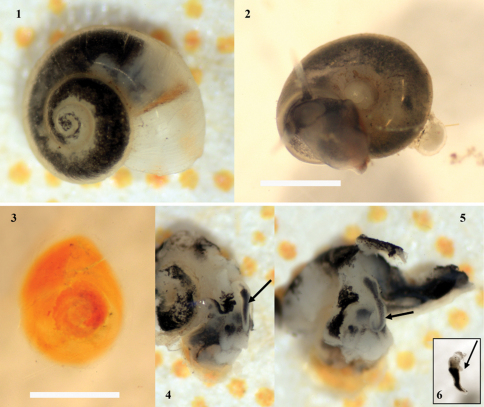
*Daphniola eptalophos* sp n. photographed in ethanol. Apical view **1**, alive specimen carrying egg capsules with an embryo on last body whorl and inside umbilicus (photographed in water) **2** operculum **3** soft body, head with tentacles and penis in situ **4-5** penis **6**. A background square represents 1 mm^2^ in Figs 1, 4, 5. Scale bar 1 mm and 0.5 mm in Figs 2 and 3 respectively. Black arrow points the penis in Figs 4-5 and the outgrowth of penis in Fig. 6.

Figs 1-6 7-10 11 14 Tables 1 2

#### Diagnosis.

Shell valvatiform to planispiral; operculum circular to ovate without peg, paucispiral with subcentral nucleus; umbilicus open and very wide; male genitalia with a slender black penis having a colorless outgrowth located near its base; female genitalia with a well-developed bursa copulatrix and two rather small receptaculum seminis.

#### Description. 

**Figures 7-10.**
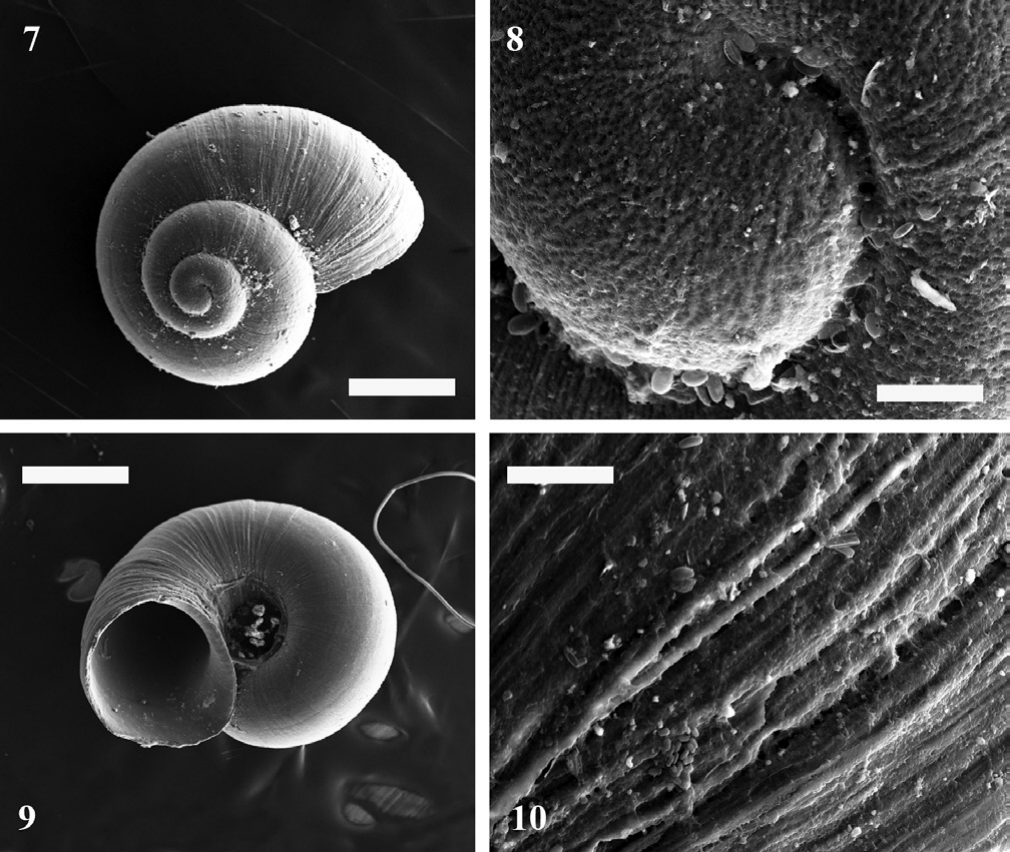
*Daphniola eptalophos* sp. n. shell images from SEM Shell habitus **7**, **8** protoconch **9** teleoconch **10** Scale bar 0.5 mm in Figs 7, 9 and 0.05 mm in Figs 8,10.

Shell minute (Tab. 1), valvatiform to planispiral, light horn-colored to whitish, transparent, glossy, finely striated (Figs 1, 7, 9).

Protoconch microsculpture composed of a dense net of irregularly shaped pores (Fig. 8), teleoconch with fine pores among the growth lines (Fig. 10).

Spire very low and blunt; 3-3.5 convex whorls, regularly growing, divided by a moderately deep suture, last whorl strongly developed.

Umbilicus open and very wide, the earlier whorls being visible inside.

Aperture prosocline, almost circular with a sharp continuous peristome and thin margins, the upper part of columellar margin slightly leaned against to the shell wall, the outer margin simple.

Operculum (Fig. 3) ovate, dark orange, thin, thicker and more colored at the nucleus, thinner and colorless at the edges, circular to ovate with weakly convex inner face, paucispiral with subcentral nucleus without any outgrowth on inner face.

In living specimens epithelium of mantle darkly grey-black pigmented, the color being clearly visible under the transparent shell, head grey-black pigmented, large eye spots present and tentacles with a median longitudinal black stripe up to the half of their length.

Penis (Figs 4-6) black pigmented except the apex and the base, long, slender, gradually tapered towards the tip with a prolonged pointed apex, sometimes like an awl (Fig. 5), with a small unpigmented outgrowth on left side near its base (Fig. 6). Occasionally, this outgrowth is not well visible.

**Figures 11-13.**
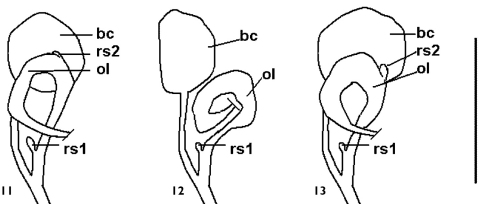
Female genitalia of *Daphniola* species.*Daphniola eptalophos* sp. n. female genitalia drawn from the only one female individual found among dissected specimens **11** *Daphniola louisi*female genitalia re-drawn from Falniowski and Szarowska (2000) **12** *Daphniola exigua*female genitalia re-drawn from Radoman (1983) **13** Scale bar 0.5 mm.

Bursa copulatrix ovate and well-developed, renal oviduct developed and unpigmented. Receptaculum seminis rs_1 _rather small, receptaculum seminis rs_2_ somewhat vestigial (Fig. 11).

#### Types.

Holotype, shell height 0.90 mm, shell width 1.50 mm, aperture height 0.70 mm, aperture width 0.60 mm, collected alive (March 18, 2011), preserved in ethanol and deposited in ZMUA 4087. Paratypes 1-2, 1: shell height 1.00 mm, shell width 1.35 mm, aperture height 0.60 mm, aperture width 0.60 mm, 2: shell height 1.10 mm, shell width 1.40 mm, aperture height 0.65 mm, aperture width 0.65 mm, collected alive (March 18, 2011), preserved in ethanol and deposited in ZMUA 4088.

#### Type locality.

 Agoriani (Eptalophos), mountain Parnassos, Sterea Ellada, Greece, 22°3013.5"N, 38°35'35.5"W, 950 m a.s.l. All the specimens were found on the surface of small stones and dead leaves accumulated on the bottom of a spring covered by a thick snow layer. None other freshwater gastropod species was found to share the same spring.

#### Further localities.

 Known only from Agoriani (Eptalophos), Sterea Ellada, Greece.

#### Etymology. 

The specific name is a noun in apposition taken from the type locality.

## Discussion

The new species collected in the Parnassos Mts. belongs to the genus *Daphniola* because it has the characteristics of this genus as defined by Radoman (1973), Schütt (1980), Radoman (1983) and Bodon et al. (2001): 1) shell very small valvatiform 2) operculum without peg 3) penis narrow, slender and elongated with a prominent apex and one outgrowth on left side 4) female genitalia with two seminal receptacles, oviduct loop and ovate bursa copulatrix well-developed.

The macrosculpture of protoconch and teleoconch of *Daphniola eptalophos* is quite similar to those described by Szarowska (2006) and Falniowski et al. (2007) for *Daphniola exigua* and *Daphniola louisi* respectively.

The shell shape of *Daphniola eptalophos* resembles that of *Daphniola hadei* (Figs 14-16, Falniowski and Szarowska 2011, page 133, Fig. 2-7), and its operculum resembles that of *Daphniola exigua* depicted by Bodon et al. (2001: page 108, Fig. 10).

**Figures 14-16.**
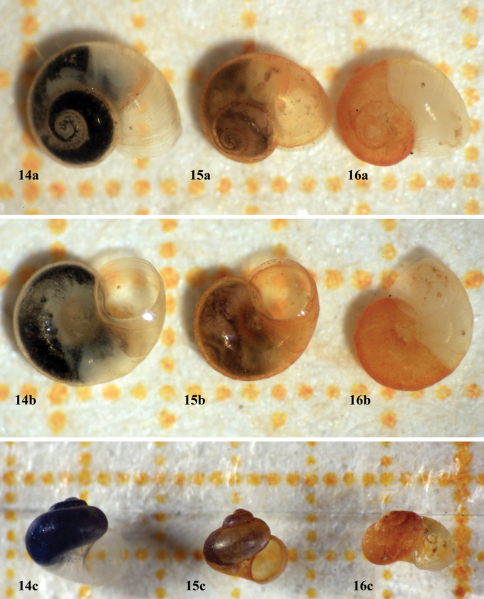
Shells of *Daphniola* species. a, apical view, b, ventral view, c. frontal view *Daphniola eptalophos* sp. n. (Agoriani) **14** *Daphniola exigua* (Marathonas, Attica) **15** *Daphniola louisi* (Kessariani, Attica) **16** A background square represents 1 mm^2^.

Several characteristics differentiate *Daphniola eptalophos* from the other known species of this genus, i.e. *Daphniola exigua* and *Daphniola louisi* and *Daphniola hadei*.

The shell of *Daphniola eptalophos* is light horn-colored to whitish in contrast to the shell of *Daphniola louisi*, which is brightly yellowish (Falniowski and Szarowska 2000), and of *Daphniola exigua*, which is whitish to greenish-whitish (Schütt 1962).

*Daphniola eptalophos* has a flatter valvatoid shell with lower spire if compared to those of *Daphniola exigua* and *Daphniola louisi* (Figs 14-16, Falniowski and Szarowska 2000). Additionally, the dimensions of its shell are different if compared to those of the other known species and subspecies of *Daphniola* (Tables 1-2).

**Table 1.** *Daphniola eptalophos* sp. n. shell morphometry. Measurements in mm. Coefficient of variation (CV) in percent =(SD*100/¯X), ¯X=mean, SD= standard deviation, n=number of specimens measured.

*Daphniola eptalophos* sp. n. n=18		**sh**	**sw**	**ah**	**aw**	**sh/sw**	**ah/aw**	**sh/ah**	**sw/aw**
	Min	0.90	1.10	0.50	0.50	0.53	0.75	1.38	1.87
	Max	1.25	1.90	0.80	0.75	0.91	1.33	2.00	3.17
	*¯X*	1.09	1.46	0.66	0.65	0.75	1.03	1.65	2.28
	SD	0.09	0.21	0.08	0.07	0.10	0.13	0.19	0.34
	CV	8.26	14.38	12.12	10.77	13.33	12.62	11.51	14.91

**Table 2.** Shell morphometry of *Daphniola* species. Measurements in mm.

***Daphniola* species **		**sh**	**sw**	**ah**	**aw**
*Daphniola louisi* Falniowski and Szarowska (2000), Falniowski et al. (2007)	Min	1.09	1.17	0.59	0.59
	Max	1.45	1.69	0.98	0.85
*Daphniola exigua* Schütt (1962)^ *^, Schütt (1980)^ **^, Radoman (1983)^ ***^, Reischütz (1984)^ ****^, Falniowski et al. (2007)	Min	0.99	1.00	0.63	0.60
	Max	1.58	1.40	0.87	0.87
*Daphniola hadei* Falniowski and Szarowska (2011)	Min	0.84	1.14	0.55	0.52
	Max	0.85	1.15	0.57	0.54
*Daphniola eptalophos* sp. n. Present study	Min	0.90	1.10	0.50	0.50
	Max	1.25	1.90	0.80	0.75

^*^As *Horatia (Horatia) exigua*, ^**^ as *Horatia (Daphniola) exigua*, ^***^ as *Daphniola graeca*, ^****^as *Horatia (Daphniola) exigua pangaea*.

The color of the operculum in *Daphniola eptalophos* is dark orange while in *Daphniola exigua* is yellowish brown (Schütt 1980) and in *Daphniola louisi* light yellowish.

The umbilicus of *Daphniola eptalophos* is open and wide such as the umbilicus of *Daphniola louisi* (Falniowski & Szarowska, 2000) and *Daphniola hadei* (Falniowski and Szarowska, 2011). In contrast, the umbilicus of *Daphniola exigua* is open but narrow (Schütt 1980; Reischütz 1984; Bodon et al. 2001) or semi-opened (Radoman 1973; Radoman 1983).

Body and head of *Daphniola eptalophos* are dark pigmented like that of *Daphniola exigua* (Falniowski et al. 2007); in *Daphniola louisi*, the pigmentation of the soft parts is limited to the delicate spots on the visceral sac covering the digestive gland and gonad while the head is unpigmented (Figs 14-16, Falniowski and Szarowska 2000). The soft body of *Daphniola hadei* is pigmentless (Falniowski and Szarowska 2011).

The eye spots of *Daphniola eptalophos* are large like in *Daphniola louisi* (Falniowski and Szarowska 2000), whereas the eye spot of *Daphniola hadei* are rather small (Falniowski and Szarowska 2011).

The penis of *Daphniola eptalophos* is more slender and elongate than that of *Daphniola louisi* (Falniowski and Szarowska 2000: page 184, Figs 18-25). *Daphniola eptalophos* differs from its congeners in the lateral outgrowth on the left side of penis: this outgrowth is small, rather triangular and located near its base in *Daphniola eptalophos*, it is long, slender and located at half the penis length in *Daphniola exigua* (Radoman 1983: page 84, Fig. 45) and it is small, blunt and located at half the penis length in *Daphniola louisi* and *Daphniola hadei* (Falniowski and Szarowska 2000: page 184, Figs 18-25, and Falniowski and Szarowska 2011: page 135, Figs 16-18). Finally, the penis of *Daphniola eptalophos* is almost entirely black pigmented, a characteristic not observed in any other *Daphniola* species.

Some of the specimens collected were observed to be carrying a single hemispherical egg capsule inside the umbilicus or attached to the body whorl with an embryo at different stage of maturation (Fig. 2). The attachment of egg capsules to the shells of the same species has not been referred in literature for any other *Daphniola* species but it has been recorded in some other hydrobiid taxa with wide umbilicus such as *Tarraconia gasulli* (Boeters, 1981) and *Boetersiella wolfi* Boeters & Glöer, 2007 (Ramos et al. 2000, Boeters and Glöer 2007 respectively).

To date, *Daphniola eptalophos* sp. n. has been found in only one spring. This fact in combination with its low population density indicates that the new species will be highly sensitive towards any kind of change of its biotope. Obviously, a monitoring of the new species is immediately required and the assessment of its population status and trends is of high priority.

Unfortunately “hydrobioid” localities in Greece, most of them springs, are prone to changes (Szarowska and Falniowski 2004, Szarowska and Falniowski 2011b) due to urbanization, water pollution, waste accumulation, tourism and agricultural practices. Many of these localities have been destroyed, and a decline or even loss of endemic hydrobiid taxa has already been recorded (Ryan and Griffiths 2001, Szarowska and Falniowski 2004, Albrecht et al. 2006, Regnier et al. 2009, Szarowska and Falniowski 2011a).

Effective conservation measures must be urgently taken to protect “hydrobioid” localities in Greece, among them the spring nearby Agoriani, before their unique gastropod fauna disappears.

### Key to the Daphniola species

**Table:** 

1	Shell valvatiform or valvatiform to planispiral, umbilicus open and wide, body unpigmented	2
–	Shell valvatiform or valvatiform to planispiral, body and head pigmented	3
2	Shell valvatiform, penis big and massive with triangular shape and a small blunt outgrowth at the middle of its length	*Daphniola louisi*
–	Shell valvatiform to planispiral, penis with long and narrow filament and a small blunt outgrowth at the middle of its length	*Daphniola hadei*
3	Shell valvatiform, umbilicus partly covered by peristome, penis pigmentless, narrow and slender with a long outgrowth at the middle of its length	*Daphniola exigua*
–	Shell valvatiform to planispiral penis very dark-colored, narrow, slender with a prolonged pointed apex and a small outgrowth near its base	*Daphniola eptalophos*

## Supplementary Material

XML Treatment for Daphniola eptalophos
